# Ral GTPase is essential for actin dynamics and Golgi apparatus distribution in mouse oocyte maturation

**DOI:** 10.1186/s13008-021-00071-y

**Published:** 2021-06-10

**Authors:** Ming-Hong Sun, Lin-Lin Hu, Chao-Ying Zhao, Xiang Lu, Yan-Ping Ren, Jun-Li Wang, Xiang-Shun Cui, Shao-Chen Sun

**Affiliations:** 1 Department of Animal Sciences, Chungbuk National University, Cheongju, 28644 South Korea; 2 The Affiliated Hospital of Youjiang Medical University for Nationalities, Baise, 533000 China; 3 College of Animal Science and Technology, Nanjing Agricultural University, Nanjing, China; 4 College of Basic Medical Sciences, Zunyi Medical University, Zunyi, China

**Keywords:** GTPase, Oocyte, Meiosis, Actin, Golgi apparatus

## Abstract

**Background:**

Ral family is a member of Ras-like GTPase superfamily, which includes RalA and RalB. RalA/B play important roles in many cell biological functions, including cytoskeleton dynamics, cell division, membrane transport, gene expression and signal transduction. However, whether RalA/B involve into the mammalian oocyte meiosis is still unclear. This study aimed to explore the roles of RalA/B during mouse oocyte maturation.

**Results:**

Our results showed that RalA/B expressed at all stages of oocyte maturation, and they were enriched at the spindle periphery area after meiosis resumption. The injection of RalA/B siRNAs into the oocytes significantly disturbed the polar body extrusion, indicating the essential roles of RalA/B for oocyte maturation. We observed that in the RalA/B knockdown oocytes the actin filament fluorescence intensity was significantly increased at the both cortex and cytoplasm, and the chromosomes were failed to locate near the cortex, indicating that RalA/B regulate actin dynamics for spindle migration in mouse oocytes. Moreover, we also found that the Golgi apparatus distribution at the spindle periphery was disturbed after RalA/B depletion.

**Conclusions:**

In summary, our results indicated that RalA/B affect actin dynamics for chromosome positioning and Golgi apparatus distribution in mouse oocytes.

## Background

The quality of oocyte maturation is one of the important factors that determine the following embryonic development. In mammals, the oocytes undergo two consecutive cell divisions during the maturation process [37]. First, the oocytes are arrested at the germinal vesicle (GV) stage in ovarian follicles that the nucleus is in the center of the oocyte at this point. After germinal vesicle breakdown (GVBD), the meiosis resumes and the meiotic spindle is anchored at or near the center of the cytoplasm during the development of oocyte to the metaphase I (MI) of the first meiosis. When the chromosomes are neatly arranged on the equatorial plate, the meiotic spindle starts to migrate along the long axis to the nearest cell cortex. After producing a haploid oocyte and a small polar body, the oocytes are arrested at metaphase II (MII) until fertilization [2]. Oocyte asymmetric division is a delicate and complex cellular process, which requires the cytoskeleton, chromosomes and various protein molecules in the oocyte to work in an orderly manner together to ensure precise regulation of the normal progress [33].

Actin filaments play critical roles in nucleus positioning, spindle migration and anchoring, long-range vesicle transport, chromosome separation and polar body extrusion during oocyte meiosis [1, 31, 34, 40]. Actin nucleators are the main regulators for the actin dynamics in mammalian oocytes. For example, Arp2/3 complex is reported to involve into actin-mediated spindle migration to the oocyte cortex [38]; while Formin 2 deficiency caused the central-arrested chromosomes in mouse oocytes [19]. Other formin subfamilies such as mDia1, Formin like 1 (FMNL1), FMNL3 and FHOD1 are also shown to affect actin assembly or distribution for spindle migration in mouse oocytes [28, 29, 47]. Besides actin nucleators, small GTPases recently are shown to be the upstream molecules for the actin nucleators. Rho GTPase RhoA-mediated ROCK-LIMK pathway and Arf GTPase Arf6 are shown to affect Arp2/3 complex or cofilin for actin assembly/disassembly in mammalian oocytes [6–8].

The Golgi apparatus plays an important role in many intracellular trafficking events, mainly including the protein modification and delivery [23]. In mouse oocytes, the Golgi apparatus go through the fragmentation after GVBD and is further fragmented and distributed around the spindle until the MII stage [26]. GM130 is a cis-Golgi protein, which can regulate microtubule and cooperate with the MAPK pathway for spindle organization, migration and asymmetric division in mouse oocyte maturation [45]. The loss of the trans-Golgi protein TGN38 would lead to an increase in metaphase I arrest, accompanied by the failure of the spindle checkpoint activation and actin cap formation, which ultimately leads to a decrease in the discharge rate of the first polar body [5].

Ral (Ras-like) family are the members of the Ras branch of the Ras superfamily of small GTPases [24]. Ral family is a kind of low molecular weight protein, which has the cycle between inactive GDP-bound and active GTP-bound states [12]. Ral has high homology with other Ras proteins, and play an important role in regulating many cell biological functions, including membrane transport, signal transduction, cell division and cytoskeletal dynamics [24, 44]. The Ral family consists of RalA and RalB, that are composed of 206 amino acids with nearly 85% of the amino acid sequence is same [3, 4]. The major differences between RalA and RalB is the C-terminal region (hypervariable region), 180 residues in RalA (181 residues in RalB) and four C-terminal residues of the membrane-bound domain [10]. The functions of Ral protein on the regulation of actin filaments have been reported in a variety of models. In *X. laevis* embryos, the interaction between active Ral and its downstream effector RalBP1 involves in endocytosis and regulates the dynamics of the actin cytoskeleton, and active Ral determines the localization and expression level of RalBP1 on the actin cytoskeleton. In HeLa cells, RalBP1 also relates to the mitotic spindle and the centrosome, a localization that could be negatively regulated by active Ral [11]. In enterocytes, the interaction of Ral-exocyst is also related to the membrane nanotube formation, which is a new type of cell–cell communication that based on the formation of actin thin-membranous nanotubes between adjacent cells [15]. RalA has been reported to be a signal transduction intermediate product that regulates the actin cytoskeleton. GTP-RalA could elicit actin-rich filopods on the surface of Swiss 3T3 cells and recruits filamin into the filopodial cytoskeleton [27]. In Drosophila oogenesis, the expression of dominant negative RalA disrupted the migration and assembly of border cell and led to defects in oocyte polarization [14, 21]. However, the function of RalA/B in meiosis of mammalian oocytes have not been studied.

In this study, we adopted the knock down approach and explored the role of RalA/B in the process of mouse oocyte maturation by injection RalA/B siRNAs. Our results showed that the perturbation of RalA/B affected chromosome positioning and Golgi apparatus distribution by regulating actin dynamics, which further led to the failure of polar body extrusion during mouse oocyte meiosis.

## Results

### Expression and subcellular localization of RalA/B during mouse oocyte meiosis

**Fig. 1:**
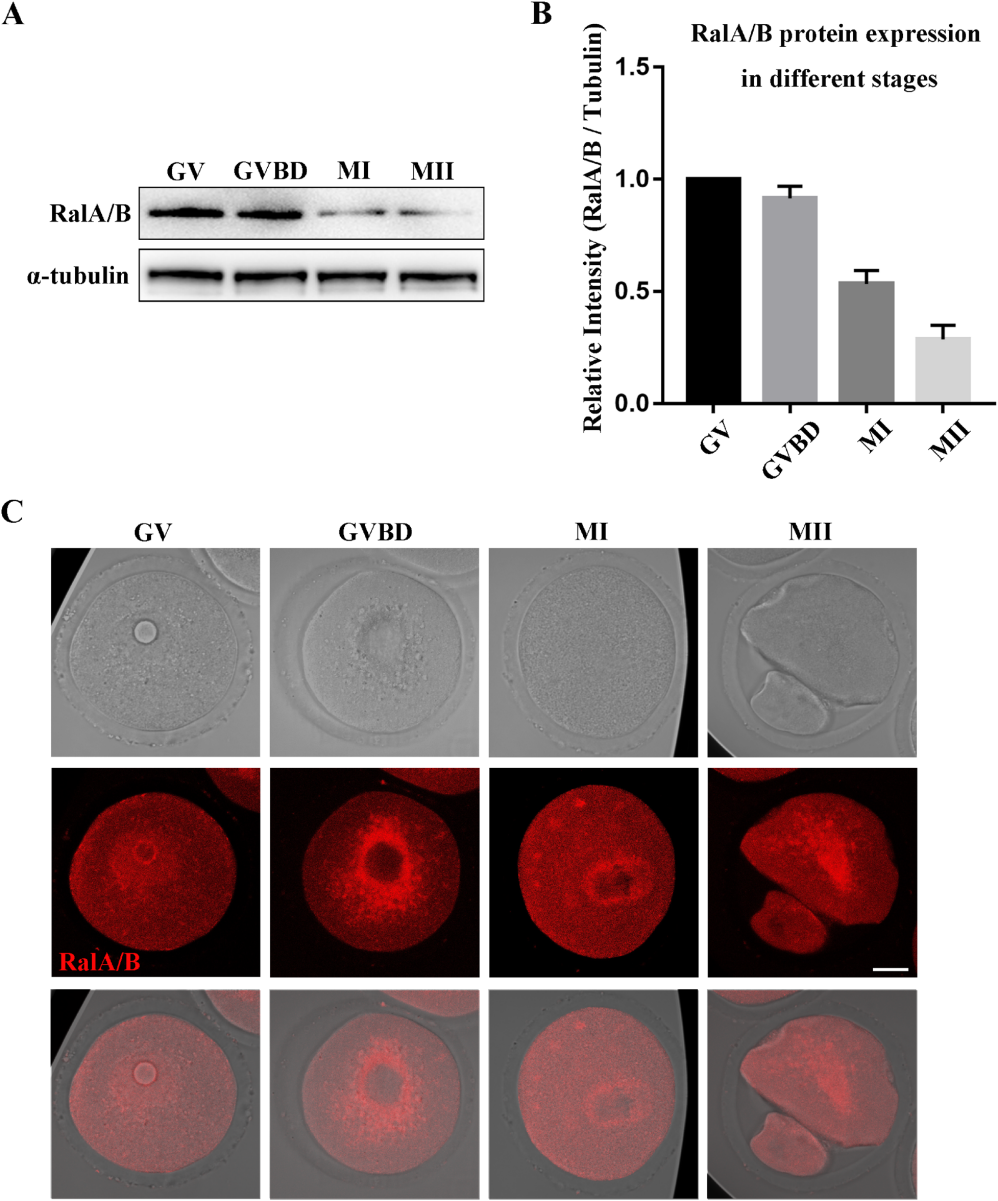
Subcellular localization of RalA/B during mouse oocyte meiosis. **A** Results of the expression of RalA/B protein in different stages by Western blotting. **B** The band intensity analysis for the RalA/B. RalA/B were all expressed at GV, GVBD, MI and MII stages. **C** Subcellular RalA/B localization during mouse oocyte meiosis based on staining with an anti-RalA/B antibody. In GV stage, RalA/B were enriched inside the germinal vesicle. After GVBD, RalA/B were mainly around the chromosomes. When oocytes were in the MI and MII stages, RalA/B were mainly distributed around the spindle. Red, RalA/B. Bar = 20 µm. *P < 0.05, **P < 0.01

In order to explore the role of RalA/B in the meiosis process of mouse oocytes, we first detected the expression and localization of RalA/B at all stages of oocytes by Western blotting and immunofluorescence staining. We cultured oocytes for 0, 4, 9, and 12 h, corresponding to vesicles (GV), germinal vesicle breakdown (GVBD), metaphase I (MI) and metaphase II (MII) during meiosis, respectively. As shown in Fig. 1A, RalA/B were expressed at all stages of oocyte maturation. Moreover, the results of densitometry analysis showed that the expression of RalA/B gradually decreased during the meiosis I of mouse oocytes (Fig. 1B). Next, the oocytes were stained with RalA/B antibody to detect their localization. As shown in Fig. 1C, in GV stage, RalA/B were enriched inside the germinal vesicle. After GVBD, RalA/B were mainly located around the chromosomes. When oocytes were in the MI and MII stages, RalA/B were mainly distributed around the spindle. The expression and localization of RalA/B in oocyte indicate that RalA/B play important roles during meiotic maturation.

### Knockdown of RalA/B affects mouse oocyte polar body extrusion

**Fig. 2:**
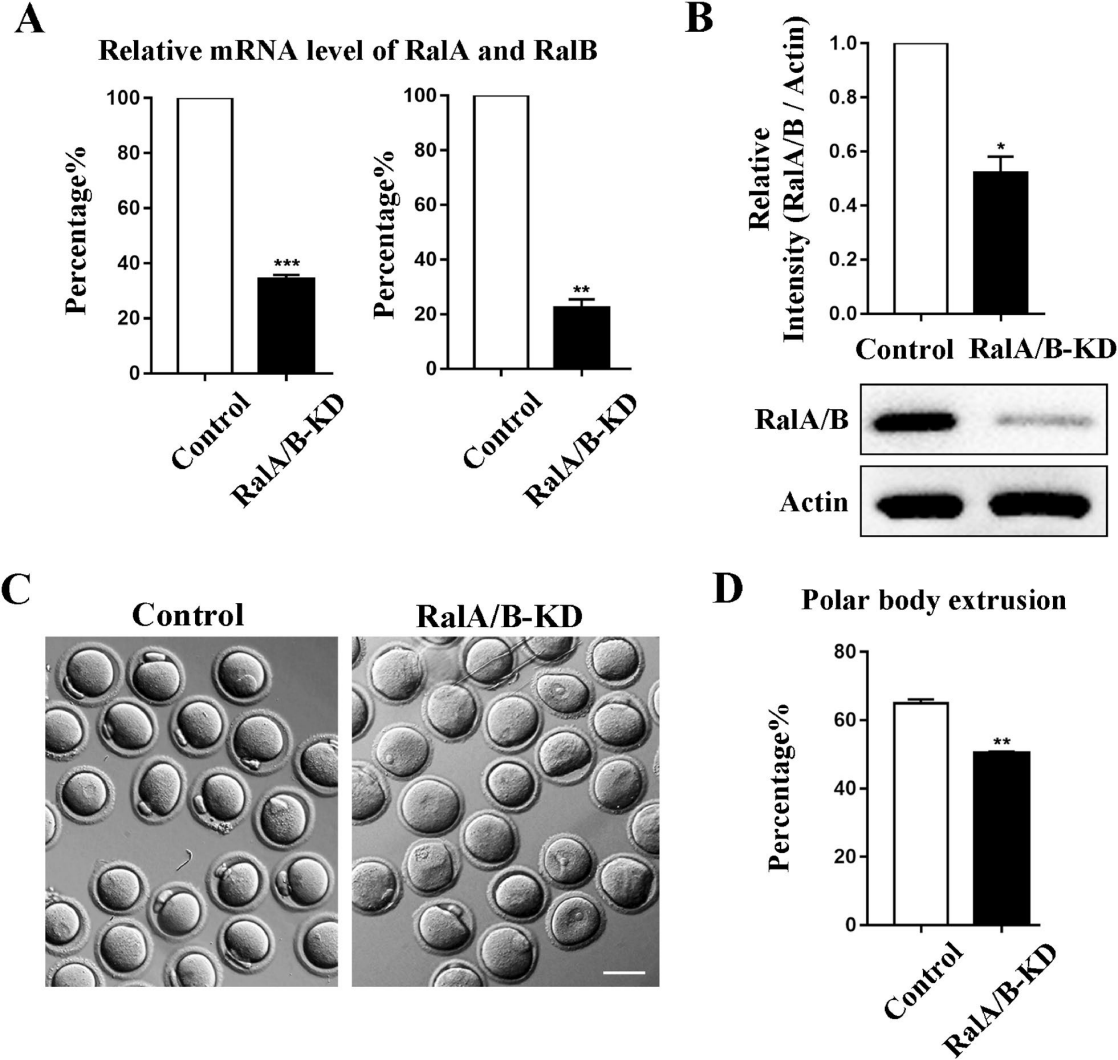
Knockdown of RalA/B affects mouse oocyte maturation. **A** Real-time quantitative PCR result of RalA and RalB mRNAs expression level in RalA/B-KD group and control group. The relative mRNAs expression of RalA and RalB were significantly decreased in RalA/B-KD group. *P < 0.05.**P < 0.01, ***P < 0.001. **B** Western blot result of the protein expression of RalA/B after RalA/B siRNAs injection. The band intensity analysis for the RalA/B after RalA/B siRNAs injection. Compared with the control group, the protein expression of RalA/B was significantly decreased in RalA/B-KD group. *P < 0.05. **C** The typical picture for the oocytes polar body extrusion after RalA/B siRNAs injection. Bar = 80 µm. **D** The rate of polar body extrusion after RalA/B siRNAs injection. The rate of polar body extrusion was significantly reduced after RalA/B siRNAs injection. *P < 0.05

To investigate the potential functions of RalA/B in the process of oocyte meiosis maturation, we employed microinjection of siRNAs to knockdown the expression of RalA/B protein and used real-time quantitative PCR to detect the knockdown efficiency. As shown in Fig. 2A, after injection of RalA/B siRNAs, the relative expression levels of RalA and RalB mRNA in RalA/B-KD group oocytes were significantly reduced compared with the control group (RalA: control group, 100.00%, n = 120; RalA/B-KD group, 34.08 ± 1.68%, n = 120, P < 0.001. RalB: control group, 100.00% 0, n = 120; RalA/B-KD group, 22.28 ± 3.14%, n = 120, P < 0.01). Using western blotting, it was found that the expression of RalA/B protein in the RalA/B-KD group was significantly decreased, and the results of densitometry analysis were also consistent with this (control group, 1.00 ± 0.00, n = 380; RalA/B-KD group, 0.52 ± 0.06, n = 380, P < 0.05) (Fig. 2B). We then cultured the oocytes for 12 h after RalA/B knockdown to examine the oocyte polar body extrusion, and we observed that oocyte maturation was disturbed: most oocytes extruded the first polar body in the control group while a big proportion of oocytes failed to develop to MII stage in the RalA/B-KD group (Fig. 2C). The rate of polar body extrusion of the two groups were analyzed respectively, and we found that compared with the control group, the rate of polar body extrusion in RalA/B-KD group was significantly reduced (control group, 64.92 ± 1.17%, n = 162; RalA/B-KD group, 50.33 ± 0.48%, n = 205, P < 0.01) (Fig. 2D). Therefore, the results indicate that the knockdown of RalA/B lead to the failure of oocyte maturation.

### Knockdown of RalA/B increases the actin density in mouse oocytes

**Fig. 3:**
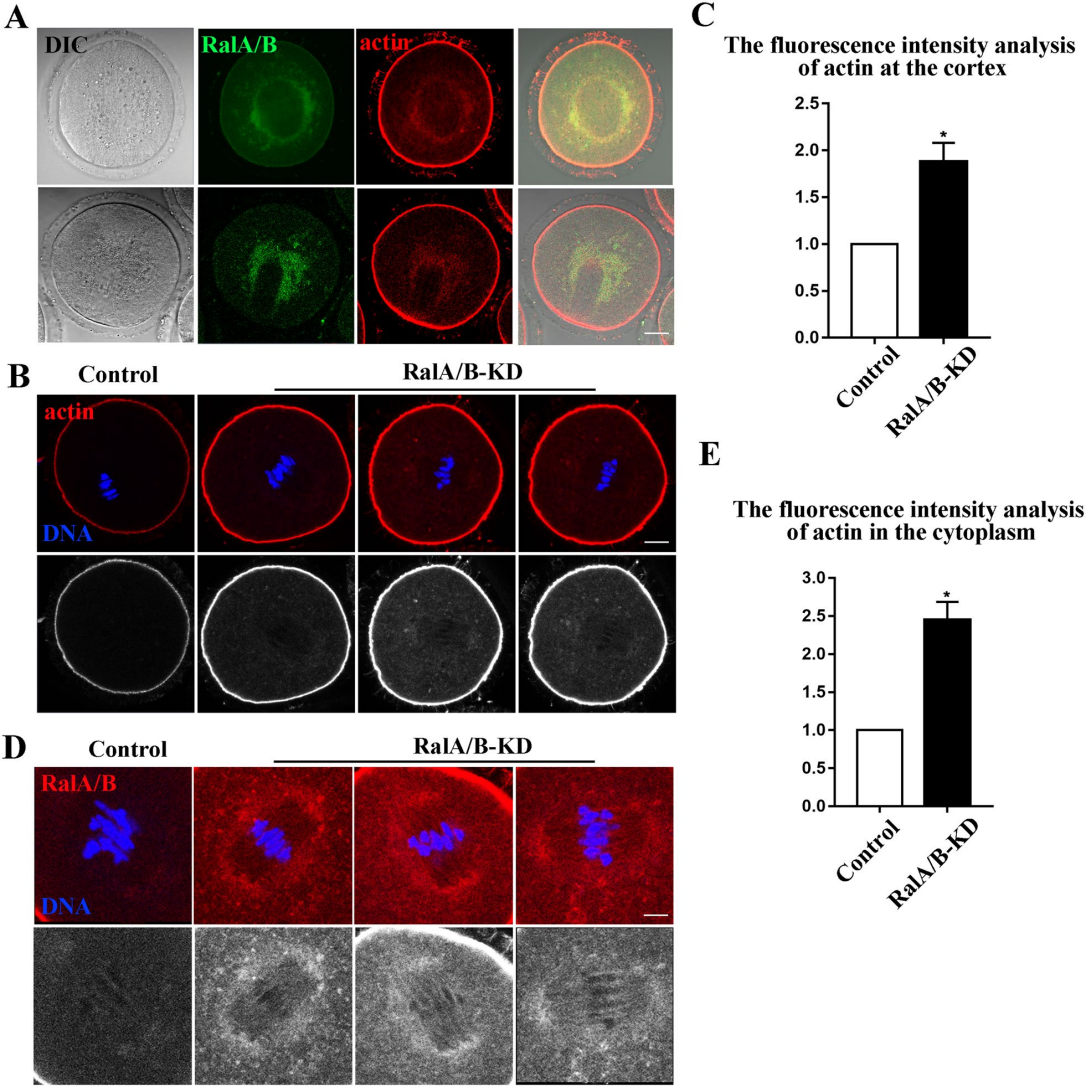
Knockdown of RalA/B increases the actin density in mouse oocytes. **A** The typical picture of RalA/B and actin localization. RalA/B showed similar localization pattern with actin in the MI stage oocytes. DIC, differential interference contrast. Red, actin; Green, RalA/B; Bar = 20 μm. **B** The typical picture for the intensity of actin at cortex after RalA/B siRNAs injection. Red, actin. Blue, DNA. Bar = 20 µm. **C** The fluorescence intensity of actin at the cortex after RalA/B siRNAs injection. Compared with the control group, the relative fluorescence intensity of actin at cortex was significantly increased in RalA/B-KD group. *P < 0.05. **D** The typical picture for the intensity of actin in the cytoplasm after RalA/B siRNAs injection. Red, actin. Blue, DNA. Bar = 5 µm. **E** The fluorescence intensity of actin in the cytoplasm after RalA/B siRNAs injection. Compared with the control group, the relative fluorescence intensity of actin in the cytoplasm was significantly increased in RalA/B-KD group. *P < 0.05

Since the localization of RalA/B is similar with the actin filaments in the oocyte cytoplasm (Fig. 3A), we next examined the effects of RalA/B on the distribution and assembly of actin. We employed F-actin staining with phalloidin to observe the distribution of actin both in the membrane and cytoplasm of MI mouse oocytes. As shown in Fig. 3B, after RalA/B siRNAs injection, the accumulation of actin fluorescence signal at the cortex was significantly increased compared with that of the control group. The result of fluorescence intensity analysis was also consistent with this (control group, 1.00 ± 0.00, n = 66; RalA/B-KD group, 1.88 ± 0.20, n = 68, P < 0.05) (Fig. 3C). In addition, our study also showed that the fluorescence intensity of cytoplasmic actin was also significantly increased after RalA/B siRNAs injection (Fig. 3D). Fluorescence intensity analysis also confirmed this (control group, 1.00 ± 0.00, n = 66; RalA/B-KD group, 2.45 ± 0.24, n = 68, P < 0.05) (Fig. 3E). These results indicate that knock down of RalA/B in mouse oocytes disturbs the actin dynamics.

### Knockdown of RalA/B disrupts chromosome positioning in mouse oocytes

**Fig. 4:**
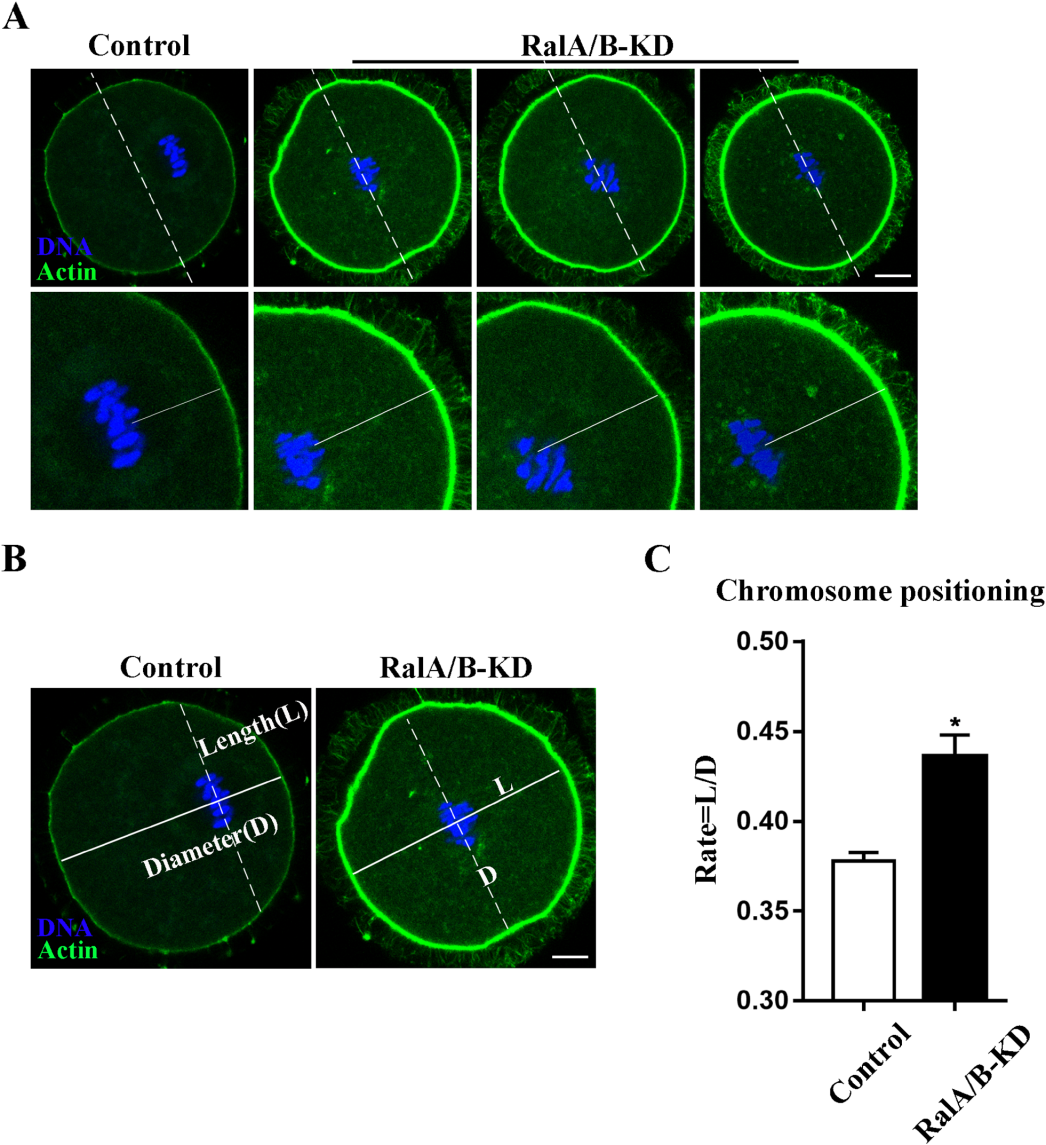
Knockdown of RalA/B disrupts chromosomes positioning during mouse oocytes meiosis. **A** The typical picture for the chromosomes position after RalA/B siRNAs injection. Green, actin. Blue, DNA. Bar = 20 µm. **B** The typical picture for the distance ratio between the central axis of the chromosomes and the cortex in oocytes after RalA/B siRNAs injection. We defined the diameter of oocyte as D and the length of the central axis of the chromosomes to oocyte cortex as L. Green, actin. Blue, DNA. Bar = 20 µm. **C** The average distance ratio after RalA/B siRNAs injection. Compared with the control group, the average distance ratio was significantly increased in RalA/B-KD group. Green, actin. Blue, DNA. Bar = 20 µm. *P < 0.05

Since the chromosomal positioning to the cortex at the late MI stage of mammalian oocytes is mediated by F-actin, we further explored the distance of chromosomes to the cortex in oocytes of the control group and RalA/B-KD group after RalA/B knockdown. As shown in Fig. 4A, we found that after injection of RalA/B siRNAs and cultured for 10 h, the chromosomes of most oocytes in the control group migrated to the cortex, while in the RalA/B-KD group, the chromosomes showed central-arrested position (Fig. 4A). In addition, we also measured the distance ratio between the central axis of the chromosomes and the cortex in oocytes (Fig. 4B). As shown in Fig. 4C, the average distance ratio in the RalA/B-KD group increased significantly compared with the control group (control group, 1.00 ± 0.00, n = 43; RalA/B-KD group, 0.77 ± 0.05, n = 46, P < 0.05) (Fig. 4C). Our results suggest that RalA/B play an important role in the positioning of chromosomes in oocytes.

### Knockdown of RalA/B disturbs Golgi apparatus distribution in mouse oocytes

**Fig. 5:**
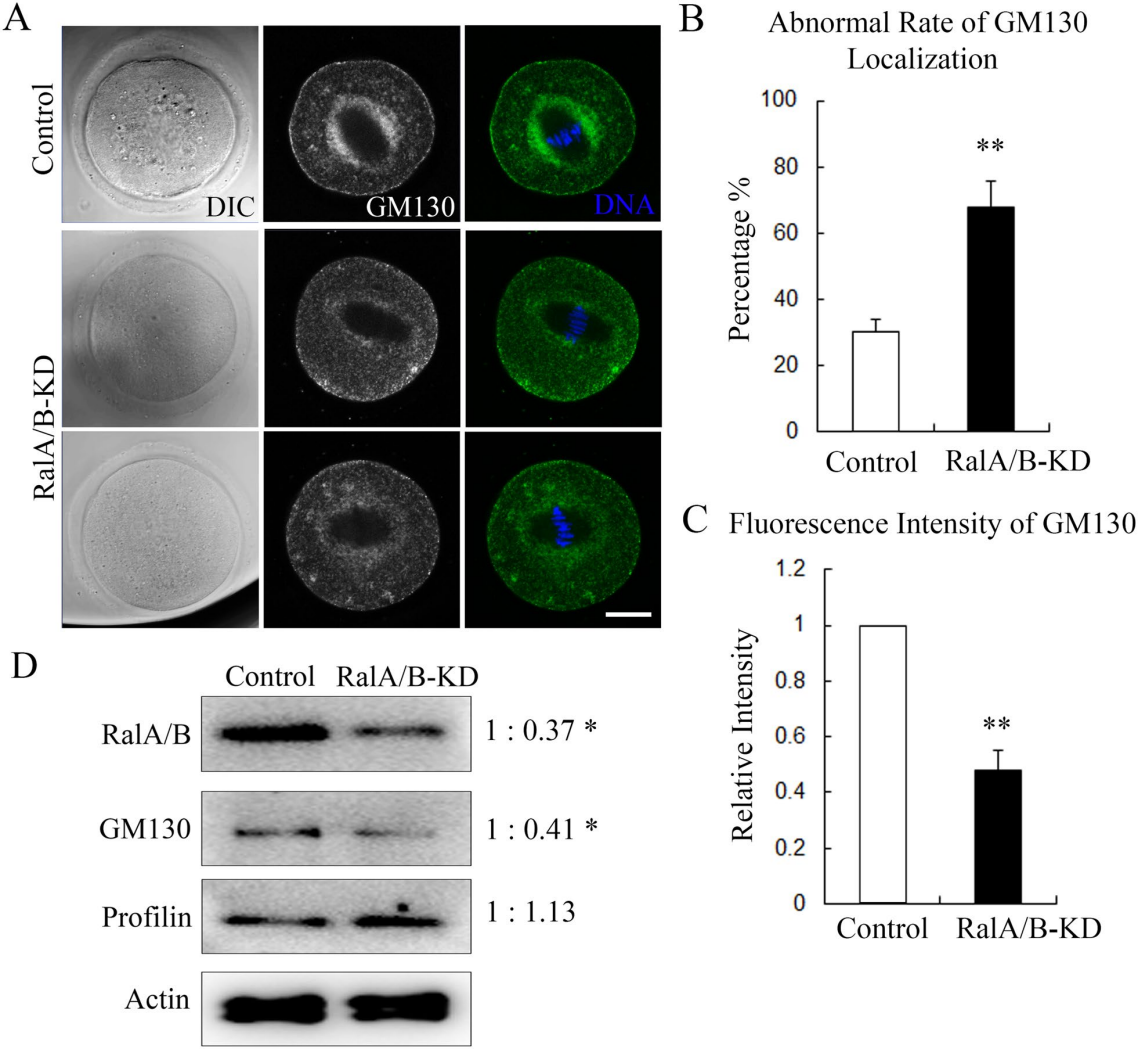
Knockdown of RalA/B disturbs Golgi apparatus distribution in mouse oocytes. **A** The typical picture for the Golgi apparatus distribution at MI stage of oocytes after RalA/B siRNAs injection. Green, GM130; blue, DNA. Bar = 20 µm. **B** The rate of abnormal GM130 localization after RalA/B siRNAs injection. **P < 0.01. **C** The relative fluorescence intensity of GM130 after RalA/B siRNAs injection. **P < 0.01. **D** The western blot results for the expression of RalA/B, GM130, Profilin after RalA/B siRNAs injection. *P < 0.05

Previous studies indicated that RalA/B were related with Golgi apparatus, we then examined the distribution of Golgi apparatus by staining GM130. As shown in Fig. 5A, in the control group, the Golgi apparatus were located at the spindle periphery in the oocytes, while the signal of GM130 was significantly decreased in the RalA/B-KD group compared with the control oocytes. The statistical analysis data showed that the abnormal rate of GM130 localization was significantly higher than the control group (30.27 ± 3.47%, n = 40 vs 67.57 ± 8.2%, n = 40; P < 0.01) (Fig. 5B), and the fluorescence intensity analysis data also confirmed this (1, n = 30 vs 0.48 ± 0.07, n = 30; P < 0.01) (Fig. 5C). We also examined the GM130 protein expression, and the results showed that after RalA/B depletion, the expression of GM130 was significantly decreased compared with the control group (Fig. 5D). These data indicate that RalA/B are essential for the Golgi apparatus distribution in the oocytes.

## Discussion

Our current study was designed to explore the functions of RalA/B in the meiosis of mouse oocytes. The results indicated that knockdown of RalA/B affected the position of chromosomes and Golgi apparatus by disrupting the distribution and assembly of actin, and ultimately lead to the failure of oocyte maturation.

RalA and RalB, as two homologous isomers of Ral family, which are found only in animal cells and are important regulators of biological processes such as cell migration, cytoskeleton dynamics, cell proliferation, apoptosis, and carcinogenesis [9]. In mouse embryonic fibroblasts, RalA and RalB both showed plasma membrane and endosome localization [13]. And in CFPAC-I cells, endogenous RalB was found to be strongly localized to the leading edge, which a specific area on the plasma membrane. Compared with RalB, the endogenous RalA localization was shown to be cytoplasmic pattern, instead of the leading edge localization [41]. Studies have also reported that endogenous RalA was found abundantly at the plasma membrane as well as throughout the cytoplasm and at internal membranes in HEK-TtH cells that stably expressed either empty vector or kinase-inactive Aurora-AK162R [22]. Our data showed that RalA/B were expressed in all developmental stages of mouse oocyte meiosis, and were mainly distributed around the spindle after GVBD, which were similar to the localization of actin filaments in the cytoplasm of mouse oocytes, indicating that RalA/B have a potential relationship with actin filaments.

To explore the potential role of RalA/B in the process of meiosis in mouse oocytes, we microinjected RalA/B siRNAs to knock down the expression of RalA/B in oocytes. After the knockdown of RalA/B, we found that perturbation of RalA/B affected the first polar body extrusion of oocytes. Studies have shown that in PC12 cells, knockdown of Ral GTPase activating protein β (RalGAPβ) could down-regulate the activity of RalA and RalB [35], and the metaphase-to-anaphase transition would be delayed when RalGAPβ is deficient in HeLa cells [32]. It has also been reported that in the process of cell death induced by cisplatin, cytoprotective effect of RalA overexpression is consistently observed. Overexpression of RalA also could induce a statistically significant difference in the cell cycle. When detecting the effect of small G protein on cycloheximide (CHX) cytotoxicity, it is found that knock down of RalA enhances CHX cytotoxicity of COS7 cells [17]. In addition, depletion of RalB inhibits cell cycle progression in a p53 and p21-dependent manner by reducing the proportion of S phase in A549 cells [39]. Overall, our results indicated that RalA/B deficiency delayed the meiosis and disturbed oocyte maturation.

For further confirm the role of RalA/B in the maturation of oocytes, based on the fact that the location of RalA/B are similar to the actin in the cytoplasm, we detected the distribution and assembly of actin in oocytes after RalA/B knockdown. Our results showed that the density of actin both in the cytoplasm and at the cortex were increased. Ral GTPases act downstream of the Ras protein and play a key role in the coordination between membrane trafficking and actin polymerization [43]. The study on RalB during the oogenesis and early development of *Xenopus* showed that the embryonic cortex and nuclear actin network were destroyed after the injection of mutant RalB RNAs, which led to the abnormal movement of pigment granules in the embryo [25]. Ras/RalGDS/Ral pathway was activated during *Xenopus* morphogenesis, which participated in controlling the dynamic equilibrium between F-actin and G-actin, and directly acts on F-actin. Furthermore, overexpression of activated RalB would lead to cortical F-actin disassembly [20].

In mammalian oocytes, actin directly involves in asymmetric spindle positioning and cortical polarization. It is also the main driving force of the spindle movement to ensure the asymmetric division of oocyte [42]. Furthermore, the polarized movement of the chromosomes also depends on microfilament-mediated process during the maturation of mouse oocytes [36]. We then detected the position of the chromosomes and found that most of meiotic chromosomes were arrested in the center of oocytes, indicating that the failure of spindle migration. Besides Ral GTPases, other GTPases such as Rab35, a member of Rab GTPases, has also been reported that has multiple roles in the stability of the spindle and actin-mediated spindle migration during mouse oocyte meiosis [46]. The spindle in mouse oocytes was failed to migrate without Rab11a-positive vesicles, and the spindle and chromosomes were arrested at the cytoplasmic center. Meanwhile, although the density of actin network was increased, the actin network is static [16]. This is similar with the finding in our study, the increase of actin fluorescence intensity caused by the depletion of RalA/B in oocytes may be just an increase in actin network density, but the actin dynamic is disturbed. The distribution and functions of the Golgi/centriole complex are depended on a multitude of factors, including the actin filament cytoskeleton [18]. In mouse oocytes, it is shown that Rab8 mediated ROCK activity for actin assembly is essential for Golgi apparatus distribution [30]. While in present study we showed that deficiency of RalA/B also caused the aberrant distribution of Golgi apparatus, indicating that besides Rab subfamily, RalA/B are also another regulator for actin-based Golgi distribution in oocytes.

## Conclusions

In summary, our results show that RalA/B depletion affects the chromosomes positioning and Golgi apparatus distribution by regulating actin dynamics during meiotic maturation of mouse oocytes.

## Materials and methods

### Ethics statement

Our study was approved by the Animal Research Committee guidelines of Nanjing Agriculture University, China. All operations related to mice were performed under the guidelines of the committee. The female 4-week-old ICR mice used in our study were fed with a regular diet and kept in a room with a constant temperature of 22 °C and an appropriate 12-h light–dark cycle. The mice were euthanized by cervical dislocation.

### Oocyte harvest and culture

The ovaries were removed from the abdomen of female ICR mice and transferred to fresh M16 medium. Only the germinal vesicle intact oocytes were collected and put in M16 covered with liquid paraffin oil, and cultured in an atmosphere at 37 °C containing 5% CO_2_ to the appropriate stages for subsequent study.

### Antibodies and chemicals

Rabbit anti-RalA/B polyclonal antibody (bs-6170R-A350) was purchased from Bioss (Beijing, China), mouse anti-RalA/B monoclonal antibody (sc-374582) was purchased from Santa Cruz (Santa Cruz, CA), Phalloidin-TRITC and Hoechst 33342 were purchased from Sigma-Aldrich Corp. (St. Louis, MO). All other chemicals and reagents were from Sigma-Aldrich Corp. unless otherwise stated.

### Microinjection of RalA/B siRNAs

To knock down the expression of endogenous RalA/B mRNA in mouse oocytes, we diluted RalA (5′-GGA CUA UGA ACC UAC CAA ATT-3′) and RalB (5′-CCC UGA CGC UUC AGU UCA UTT-3′) siRNA respectively with DEPC water to 50 μM and mixed them (1:1). Eppendorf FemtoJet (Eppendorf AG) with an inverted microscope (Olympus IX53, Japan) was used to microinject 5–10 pl RalA/B siRNAs into GV oocytes. The oocytes were moved into M16 medium containing milrinone and cultured for 20–24 h, and then washed six times with M2 medium for 3 min each. After washing, we transfered the oocytes into fresh M16 medium for culture. In the negative control group, siRNA 5′-UUC UCC GAA CGU GUC ACG UTT-3′ was injected into the oocytes.

### Western blotting

We lysed at least 120 oocytes from the control group and RalA/B-KD group with 10 μl of ice-cold Laemmli sample buffer (SDS sample buffer containing 2-mercaptoethanol). Then we heated the buffer at 100 °C for 10 min and stored at − 20 °C. The protein extracts were separated by 12% sodium lauryl sulfate–polyacrylamide gel electrophoresis (SDS-PAGE), and transferred to a polyvinylidene fluoride (PVDF) membrane (Millipore, Bedford, MA) by electroblotting. Nonspecific binding sites were blocked with TBST (Tris buffered saline tween-20) containing 5% skimmed milk powder or BSA for 1 h at room temperature. The membranes were then incubated with mouse anti-RalA/B monoclonal antibody (1:500) in blocking solution overnight at 4 °C. After washing in TBST three times (10 min each time), the membranes were incubated with Horse Radish Peroxidase (HRP)-conjugated secondary antibody (1:1000) for 1 h at room temperature. Finally, the signal detection of the bands was performed using a high-sig ECL Western blotting System (Tanon, China). The band intensity values were analyzed by Image J software.

### Real-time quantitative PCR

The knockdown efficiency of RalA/B mRNAs was analyzed by using real-time quantitative PCR. In the control and RalA/B-KD group, 40 oocytes were taken and the total RNA was extracted with Dynabears mRNA Direct Kit (Invitrogen Dynal AS, Norway), then the first-strand cDNA was synthesized with PrimeScript RT Master Mix (Takara, Japan). The cDNA fragments were amplified using Rala and Ralb primers (Rala: Forward, ATG TAC GAC GAG TTT GTA GAG GA; Reverse, CCC GCT GTA TCT AAG ATG TCG A. Ralb: Forward, GCT CCC TGG TAC TTC ACA AGG; Reverse, CAG GAT GTC TAT CTG GAC CTC TT). The relative expression of the gene was determined by the 2^−ΔΔCt^ method.

### Immunofluorescent staining and confocal microscopy

The oocytes in the control and RalA/B-KD groups were fixed for 30 min with 4% paraformaldehyde at room temperature. After permeating in 0.1% Triton X-100 (in PBS, v/v) for 20 min at room temperature, we transfered the oocytes into blocking buffer (1% bovine serum albumin supplemented with PBS) for 1 h at room temperature or overnight at 4 °C. For RalA/B staining, oocytes were incubated with Rabbit anti-RalA/B polyclonal antibody (bs-6170R-A350, 1:100) overnight at 4 °C, and then washed with washing buffer (0.1% Tween 20–10 and 0.01% Triton X-100) three times, 3 min each time. To visualize actin filaments, oocytes were incubated with Phalloidin-TRITC (5 μg/ml PBS) for 1 h at room temperature. After washing three times in the same way, we incubated the oocytes with Hoechst 33342 at room temperature for 15 min. Finally, the oocytes were mounted on glass slides and observed under a confocal laser scanning microscope (Zeiss LSM 800 META; Germany).

### Statistical analysis

At least three biological replicates were used for each experimental analysis, and each replicate was completed by an independent experiment at the different time. The data was analyzed using GraphPad Prism 5 software (GraphPad, San Diego, California), Shapiro–Wilk normality test was used to detect whether the data obeyed a normal distribution and comparative statistics were performed by independent sample *T* test. Expressed as means ± SEM. When the P < 0.05, was considered significant.

## Data Availability

All data generated or analyzed during this study are included in this published article.
